# Surveillance and Genomic Evolution of Infectious Precocity Virus (IPV) from 2011 to 2024

**DOI:** 10.3390/v17030425

**Published:** 2025-03-15

**Authors:** Chengyan Zhou, Guohao Wang, Qingqing Zhou, Fanzeng Meng, Shufang Liu, Jie Huang, Xuan Dong

**Affiliations:** 1 College of Fisheries and Life Science, Shanghai Ocean University, Shanghai 201306, China; 2 State Key Laboratory of Mariculture Biobreeding and Sustainable Goods, Yellow Sea Fisheries Research Institute, Chinese Academy of Fishery Sciences, Laboratory for Marine Fisheries Science and Food Production Processes, Qingdao Marine Science and Technology Center, Key Laboratory of Maricultural Organism Disease Control, Ministry of Agriculture and Rural Affairs, Qingdao Key Laboratory of Mariculture Epidemiology and Biosecurity, Qingdao 266071, China

**Keywords:** IPV, phylogenetic analysis, *Macrobrachium rosenbergii*, *Flaviviridae*

## Abstract

Infectious precocity virus (IPV) poses a significant economic threat to the aquaculture industry by causing sexual precocity and slow growth in *Macrobrachium rosenbergii*. In this study, we conducted an in-depth investigation into the genetic evolution of IPV from 2011 to 2024 by collecting 31 IPV variants through epidemiological surveys and public databases, including 29 variants with complete genomic sequences. The phylogenetic analysis revealed that these complete genomic sequences clustered into two distinct phylogenetic clades as follows: the Southeast Asian clade and the Chinese clade. Nucleotide and protein variation analyses demonstrated a high degree of similarity, with nucleotide identity ranging from 98.5% to 100% and protein identity from 99.4% to 100%. Further analysis of protein variations within the putative coding region identified two distinct variation patterns. The average dN/dS ratio of 0.12 highlights the strong purifying selection acting on IPV, particularly on structural proteins. In conclusion, this study significantly expands the genomic database of IPV and provides valuable insights into its genetic evolution. These findings offer critical scientific evidence to enhance detection protocols and support sustainable *M. rosenbergii* aquaculture practices.

## 1. Introduction

*Macrobrachium rosenbergii*, commonly known as giant freshwater prawn, is a major aquaculture species extensively farmed in many countries, especially China and Southeast Asia [1,2]. Since 2010, a disorder termed iron prawn syndrome (IPS) has impacted the cultivation of *M. rosenbergii*. This was characterized by sexual precocity and slow growth and has caused substantial economic losses in the aquaculture industry [3,4]. The cause of IPS remained elusive for years, complicating efforts to manage and prevent the syndrome. However, in 2021, a virus known as infectious precocity virus (IPV) was reported, confirming its association with sexual precocity in *M. rosenbergii* [5].

IPV, as a single-stranded, positive-sense RNA virus in the *Flaviviridae* family, has a genome that spans 12,630 nucleotides and includes a poly(A) tail at the 3′ end. It contains two open reading frames (ORFs), ORF1 and ORF2, separated by a 125-nucleotide intergenic region, with untranslated regions at both the 5′ (967 nt) and 3′ (540 nt) ends, the genome encodes a polyprotein facilitated by a programmed ribosomal frameshift, featuring several conserved flavivirus domains such as the RNA-directed RNA polymerase (RdRp), a trypsin-like peptidase domain, and the helicase domains [5]. These structural components highlight this virus’s similarity to other flaviviruses, albeit with distinct genetic features. The *Flaviviridae* family currently encompasses four main genera—*Pegivirus*, *Pestivirus*, *Hepacivirus*, and *Flavivirus* [6]—as well as the unclassified cluster ‘Jingmenvirus’ [7,8]. The extensive host range of *Flaviviridae*, spanning a wide array of organisms, coupled with studies on viruses identified in these diverse hosts [9,10], continues to expand our understanding of the evolutionary dynamics within this viral family [11].

To comprehensively understand the genomic characteristics and epidemiological patterns of IPV, this study conducted an extensive genetic and evolutionary analysis of variants identified from our collected samples and those obtained from public databases. This analysis aimed to clarify phylogenetic relationships and genetic lineages, providing insights into the epidemiology, genetic variation, and molecular evolution of IPV.

## 2. Materials and Methods

### 2.1. RNA Sequencing Library Collection

We selected 23 meta-transcriptomic libraries related to the IPV from a large-scale meta-transcriptomic sequencing study on economic crustaceans, for all of which the host species was *M. rosenbergii* [12]. These libraries were sourced from Zhejiang, Jiangsu, Shandong, and Guangdong provinces in China. Additionally, we obtained and sequenced two extra samples from Southeast Asia using the lncRNA library preparation and Illumina NovaSeq 6000 sequencing platform by Novogene (Tianjin, China). Moreover, we also sourced 46 public RNA-sequence libraries from NCBI, which are related to studies on growth, development, and sexual precocity. Among them, only the MNON and MNOP libraries originated from *M. nipponense*, while the others were derived from *M. rosenbergii*, with contributions from China (*N* = 21) [13,14,15], Thailand (*N* = 15) [16,17], Malaysia (*N* = 6) [18], and India (*N* = 4). A total of 71 libraries were analyzed, 27 had reads mapping to IPV sequences (Virus Reads > 1), as outlined in Supplementary Table S1.

### 2.2. Sources of IPV Variants

For the meta-transcriptomic library, quality control was conducted using Fastp v0.20.0 (HaploX Biotechnology Co., LTD, Shenzhen, China, 2018) [19], and clean reads were de novo assembled using Trinity v2.15.1 with the default settings [20], as employed in our previous studies. All assembled contigs were initially screened using BLASTx against the non-redundant protein database (NR) from GenBank, downloaded on 22 February 2022, to remove sequences unrelated to viruses, including the host, plant, bacterial, and fungal sequences, and to exclude false positives with a cutoff E-value of 1 × 10^−5^. Subsequently, potential viral contigs identified in this step were annotated using BLASTn against the non-redundant nucleotide database (NT) from GenBank to confirm their viral origins. For the two newly sequenced Southeast Asian libraries, a reference-based assembly approach was adopted, aligning reads to the IPV genome (GenBank: MT084113.1) using Bowtie2-2.3.5.1-Linux-x86_64 [21], with the alignment conducted in “local” mode. Samtools v1.9 and Bcftools v1.9 [22] were then used to map the reads and assemble the genomes of the IPV variants. The number of reads mapped to each target genome is detailed in the “Virus Reads” column of Supplementary Table S1. Furthermore, we acquired four publicly accessible variants of IPV/Macrobrachium flavivirus 1 from NCBI. The GenBank accession numbers for these variants are MT084113.1, ON382579.1, MT648663.1, and MT648664.1.

(1) $$FPKM=\frac{Genome\;reads}{Total\;reads\left(Millions\right)\times Genome\;length(KB)}$$

### 2.3. Phylogenetic Analyses

To infer the evolutionary history of all IPV variants, we conducted a phylogenetic analysis using the complete genomic sequences of the viruses. Multiple sequence alignments were performed using MAFFT v7.526 [23]. Non-conserved amino acid regions were removed using Trimal v1.2 [24] with the “-automated1” parameter. The construction of the phylogenetic trees was carried out using IQ-TREE2 v2.3.5 [25]. For optimal substitution model selection, we employed an automatic model selection feature that recommends the most suitable model by comparing the fit of different models. We set up 1000 bootstrap replicates to assess the support of the tree. In addition, regions containing polymorphic variation sites were extracted, and phylogenetic trees based on these variation sites were constructed using the same method.

### 2.4. Genetic Variance Analyses

We aligned the complete genomic sequences of all IPV variants and the 3707 amino acid polyproteins produced by frameshift translation [5] using MAFFT v7.526 [23]. To analyze the genetic variance among different IPV variants, we globally visualized amino acid conservation based on the MR2018 variant using the ggmsa v1.10.0 [26] package in R v4.3.2. We performed the sequence similarity plotting of complete genomic sequences of all IPV variants using Simplot to assess the degree of similarity among the variants [27]. We also highlighted the regions with the highest density of variations within the non-structured region and the key flavivirus-related domains as follows: the flavivirus RdRp (Flavi_NS5; pfam00972; aa positions 3135 to 3655), the flavivirus DEAD domain (Flavi_DEAD; pfam07652; aa positions 2119 to 2217), the trypsin-like peptidase domain (Trypsin_2; pfam13365; aa positions 1968 to 2088), the DEXH-box helicase domain from the NS3 protease helicase (DEXHc_viral_Ns3; cd17931; aa positions 2123 to 2220), and the C-terminal helicase domain of the viral helicase (SF2_C_viral; cl18806; aa positions 2285 to 2382). Additionally, we visualized these flavivirus-related domains using the ggplot2 v3.5.1 [28] package in R v4.3.2.

### 2.5. Selection Pressure Analysis

To investigate the selection pressures acting on the coding regions of the complete genomic sequences of IPV variants, we employed the Fixed Effects Likelihood (FEL) method on the Datamonkey2.0 web server, https://www.datamonkey.org/ (accessed on 10 January 2025) [29]. FEL is a codon-based maximum likelihood approach designed to estimate the synonymous (α) and non-synonymous (β) substitution rates at each codon position, enabling the identification of sites under positive or purifying selection. For the analysis, we concatenated the ORF1 and ORF2 regions of the 29 variants and introduced a thymine (T) nucleotide at the frameshift position to generate a 3707aa coding sequence as input. FEL analysis was performed using Datamonkey’s default parameters, independently estimating synonymous and non-synonymous substitutions for each codon site. The results were visualized by plotting the substitution rates (α and β) and statistical significance values along the codon positions on the *x*-axis. Additionally, a histogram was generated to depict the distribution of dN/dS values across all codons.

## 3. Results

### 3.1. IPV Variant Dataset

Our dataset comprises 31 IPV variants, including 25 genome sequences that were assembled from samples we collected through meta-transcriptomic library construction and sequencing. Among these, 23 are whole genomes with FPKM ranging from 4.74 × 10^1^ to 4.09 × 10^6^, and two are partial genomes 01/JS/201806 and 02/JS/201806, with FPKM values of 8.03 × 10^−1^ and 8.85 × 10^−1^. These samples were collected over the period from 2018 to 2024. Additionally, two variants from Shanghai, China, were assembled from the RNA sequencing libraries available in the NCBI database, exhibiting FPKM of 4.18 × 10^2^ and 1.20 × 10^3^. The dataset is further enriched by four publicly available IPV variants from NCBI. Overall, the samples from which all the variants were derived were collected over a period from 2011 to 2024 (Table 1).

### 3.2. Phylogenetic Analyses in IPV Variants

**Figure 1:**
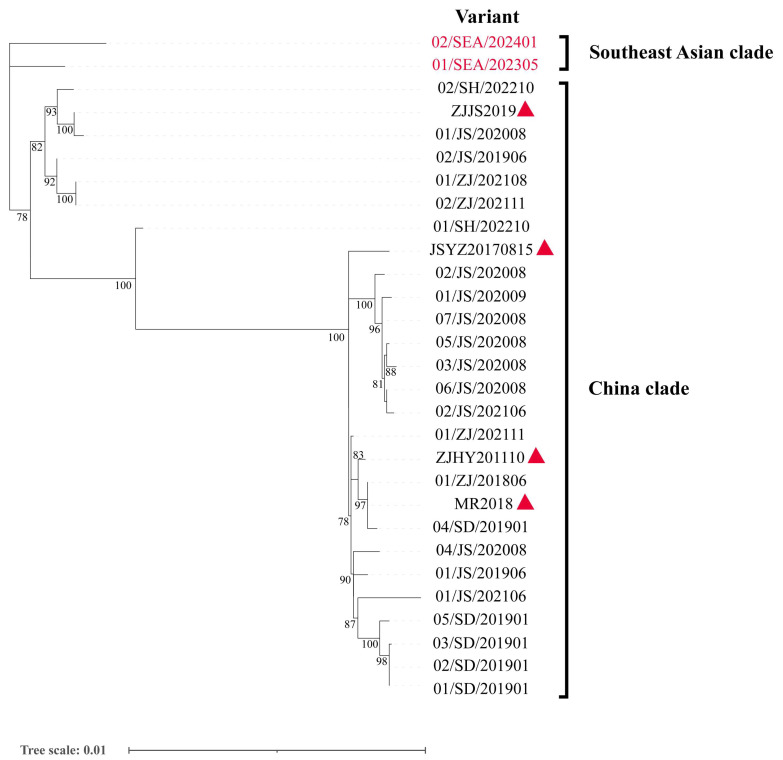
Phylogenetic analyses of 29 infectious precocity virus (IPV) variants. The analysis was conducted using the complete sequences of the 29 variants with IQ-TREE2 v2.3.5, which determined TN+F+I as the best-fit model. The scale bar indicates the number of nucleotide substitutions per site. Only bootstrap support values greater than 70% are displayed. The red font highlights the two variants from Southeast Asia, and the red triangles represent the four IPV variants currently available in NCBI with GenBank accessions MT084113.1, ON382579.1, MT648663.1, and MT648664.1.

The phylogenetic analysis in this study classified the 29 complete genomic sequences of IPV variants into two phylogenetic lineages as follows: the Southeast Asian clade and the Chinese clade (Figure 1). The Southeast Asian clade includes two variants from Southeast Asia, 01/SEA/202305 and 02/SEA/202401, which are distinctly separated from the Chinese IPV variants, forming an independent clade. The Chinese clade contains the remaining variants from different provinces of China. Although these variants were collected from different years and locations, they clustered into different groups based on genetic similarity. Notably, the variants 01/SH/202210 and JSYZ20170815, assembled from public databases, formed distinct branches. Additionally, among the 12 samples collected from Jiangsu Province between 2019 and 2021, seven clustered together in the same branch, reflecting their high genetic similarity, while the remaining samples clustered into other branches.

### 3.3. Genetic Variation in IPV Variants

**Figure 2:**
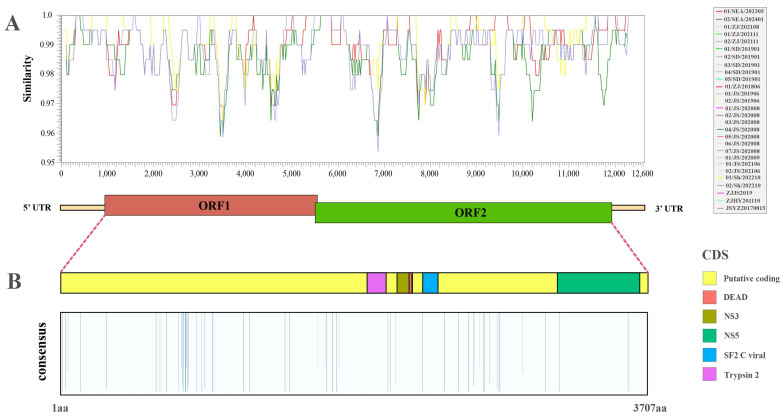
Overview of genetic variance of the 29 IPV variants. (**A**) Nucleotide similarity among the 29 variants, with the genomic structure and similarity measured relative to variant “MR2018” (12,630 nt). (**B**) Amino acid conservation among the 29 variants. The highlighted bars indicate the percentage of variants sharing the same amino acid at each position with variant “MR2018”. The bars represent 100% similarity and are downplayed for clarity. Colored boxes denote protein domains.

A comprehensive sequence alignment analysis of the genome sequences and the amino acid polyproteins resulting from frameshift translation across all variants revealed that the genomic sequence identities ranged between 98.5% and 100%, while the amino acid sequence identities ranged between 99.35% and 100% (Figure S1). For the remaining variants, the genomic sequence similarity relative to the reference variant “MR2018” ranged from 95.40% to 100% (Figure 2A). Based on the characterized amino acid variation sites, the highlighted regions (where similarity is less than 100%) were primarily concentrated in the putative coding region. However, the Trypsin_2 domain (aa positions 1968 to 2088), Flavi_DEAD and DEXHc_viral_Ns3 domains (aa positions 2119 to 2220), and the SF2_C_viral domain (aa positions 2285 to 2382) showed 100% similarity across all variants. Notably, sites with similarities less than 100% were identified only in the NS5 domain, which encodes the RdRp of flaviviruses (Figure 2B). Subsequent domain-specific amino acid alignments demonstrated that, apart from the NS5 domain, only variants 04/JS/202008 and 01/JS/202008 exhibited single amino acid variation, while all other positions remained conserved across all variants (Figures S2–S5).

### 3.4. Amino Acid Polymorphism in IPV Variants

**Figure 3:**
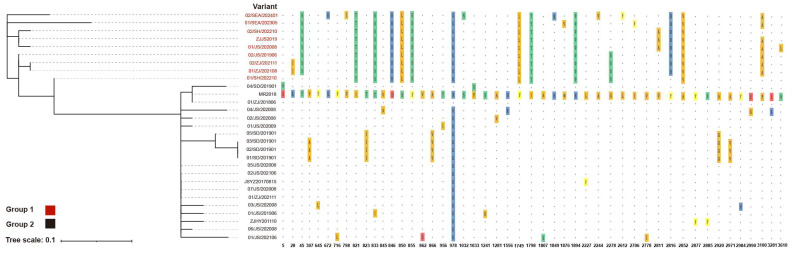
Phylogenetic tree of the 29 IPV variants based on 48 polymorphic sites. The left side shows a phylogenetic tree based on 48 polymorphic sites, while the right side presents the corresponding amino acid polymorphism information. The MR2018 sequence is used as the reference, with identical amino acids represented by “.”, and polymorphic sites highlighted with colored backgrounds. Variant names are labeled on the left side of the phylogenetic tree and the polymorphism chart, while amino acid positions are indicated below the polymorphism chart.

A total of 48 polymorphic sites were identified across 29 IPV variants, with two located within the flavivirus RdRp region (Figure S5) and 46 inconsistencies observed within the putative coding region (Figure 3). The phylogenetic analysis based on these 48 polymorphic sites revealed distinct clustering patterns, with 9 variants exhibiting unique mutations at key positions (821, 833, 846, 850, 855, 978, 1749, 1798, 1894, and 2852), differentiating them from the remaining 20 variants, leading to their classification into Group 1 and Group 2. In the RdRp region, the polymorphic site at position 3281 was exclusive to Group 2, while position 3610 was specific to Group 1. Variant 01/SH/202210, classified within Group 1, exhibited transitional evolutionary features between Group 1 and Group 2. It carried key mutations defining Group 1 but retained conserved sites of Group 2 at positions 5, 2816, and 3100. Similarly, 02/SH/202210 shared the conserved site at position 3100 with Group 2. Within Group 1, variants 01/SEA/202305 and 02/SEA/202401 exhibited two and six unique single amino acid mutations, respectively, and did not cluster with other branches. In Group 2, variants MR2018, 04/SD/201901, and 01/ZJ/201806 displayed a unique mutation at position 978, distinguishing them from all other variants. Furthermore, 01/SD/201901–03/SD/201901 and 05/SD/201901 shared five common mutations that set them apart from the remaining variants.

### 3.5. Selection Pressure in IPV Variants

**Figure 4:**
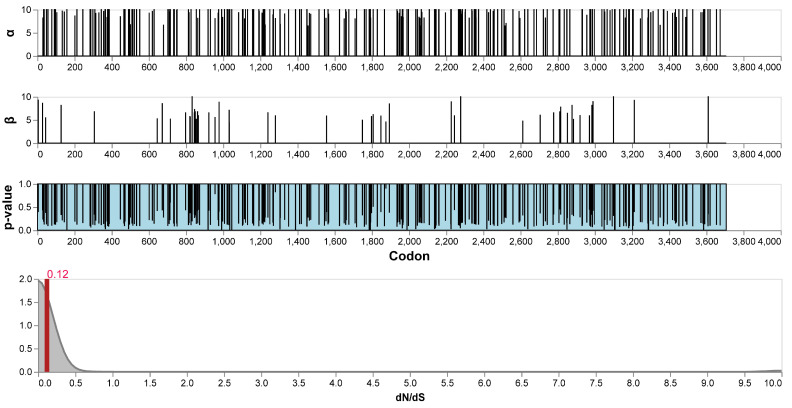
Selection pressure in the 29 IPV variants. Maximum likelihood estimates of synonymous (α) and non-synonymous rates (β) at each site. Estimates above 10 are censored at this value. *p*-values are also shown. Kernel density estimates of site-level rate estimates. The means are expressed by red rules.

Selection pressures acting on the coding sequences of 29 IPV variants were characterized using the FEL method, revealing the distribution of synonymous substitution rates (α), non-synonymous substitution rates (β), and their statistical significance (*p*-values) at each codon site (Figure 4). The results showed that most codon sites were under purifying selection (α > β), indicating that IPV has been subject to functional constraints to maintain the stability of its protein functions during evolution. However, a small number of sites exhibited significant *p*-values (*p* < 0.1), reflecting strong signals of positive selection. The distribution of dN/dS values further supported the overall selection pressure patterns across the genome. The results demonstrated that the genome is predominantly under purifying selection, with dN/dS values concentrated below 1 and a mean value of 0.12. Only a very small number of codon sites displayed dN/dS > 1, indicative of positive selection. Mapping the significant positively selected sites onto the functional domains of the polyprotein revealed that some of these sites were concentrated in the non-structural region (aa positions 800 to 900) and key functional domains of the virus, such as the RNA polymerase domain of the NS5 protein (aa positions 3135 to 3655).

## 4. Discussion

Prior to this study, only four IPV variants were publicly available [5,30], with research primarily focusing on molecular diagnostics [31,32,33] and epidemiological studies [34,35]. Preliminary analyses of the IPV genome structure were conducted [5], alongside transcriptome [36] and metabolomic analyses [37] on hosts affected by IPS to explore the mechanisms of IPS formation. It has been reported that susceptible hosts of IPV also include *M. nipponense* [34]. However, research on the origins and transmission pathways of IPV has been limited.

This study collected 27 complete IPV sequence variants from China and 2 variants from Southeast Asia, revealing two distinct phylogenetic lineages as follows: the Southeast Asian clade and the Chinese clade. Within the Southeast Asian clade, the two variants did not cluster in the same branch. In the Chinese clade, even samples collected in the same year from Jiangsu Province were distributed across different branches, clustering with variants from other times and locations. The subsequent polymorphic site analysis provided deeper insights into the molecular evolution of IPV. Based on the polymorphic sites, the variants were clearly divided into two groups, with the two Southeast Asian clade variants sharing key mutation sites with seven variants from the China clade. Interestingly, 01/SH/202210 exhibited features from both groups, suggesting that it may represent an evolutionary intermediate between the two IPV lineages, as also supported by the complete genome phylogenetic analysis. Notably, the two variants in the Southeast Asian clade harbored unique single amino acid mutations that were distinct from each other, suggesting the possibility of greater diversity in amino acid variations outside the scope of this study, potentially influenced by geographical distribution.

Although the first publicly available IPV sequence, MR2018, belongs to the Chinese clade, it exhibited a unique mutation at amino acid position 978 that distinguishes it from all other variants. Given the time elapsed between the public release of the MR2018 sequence and the outbreak of IPS, we hypothesize that during periods when IPV was not being actively monitored, viruses from diverse sources were continuously introduced and locally transmitted through multiple routes. This hypothesis is further supported by the shared unique mutation patterns observed in the four contemporaneous variants from Shandong Province, 01/SD/201901–03/SD/201901 and 05/SD/201901.

Overall, the nucleotide and protein variation analyses of these 29 complete sequence variants revealed a high level of similarity, particularly in structural proteins. However, two variants, 04/JS/202008 and 01/JS/202008, showed significant amino acid substitutions in the RdRp, with glutamic acid (E) replaced by lysine (K) and glutamine (Q) replaced by leucine (L). The observed mutations were primarily concentrated in the putative coding region, with a particularly dense cluster of variations in the 800–900 region. The selection pressure analysis further confirmed this, showing a high density of non-synonymous mutations in this region, which were consistently present in all nine variants of Group 1. This suggests that this region may have been subjected to selection pressures distinct from those experienced by other branches. Despite these localized variations, the overall IPV variants demonstrated a high degree of conservation, with an average dN/dS ratio of 0.12, indicating that these variants likely originated from a relatively recent common ancestor and have experienced strong purifying selection on structural proteins during evolution.

To explore the origin and evolution of IPV, further meta-transcriptomic surveillance of samples from regions outside China is essential. Additionally, obtaining more early-stage samples or sequencing data would greatly aid in elucidating the origin of IPV. Simultaneously, monitoring other species should be strengthened to prevent further cross-species transmission and spread of IPV.

## 5. Conclusions

In summary, our extensive phylogenetic analysis of IPV has significantly enhanced our understanding of its transmission history and supported the ongoing monitoring efforts. This study provides a scientific foundation for the improved detection and tracking of IPV, particularly in the context of international imports of *M. rosenbergii* for aquaculture. Moving forward, it is crucial to focus on *M. rosenbergii* from diverse regions affected by IPS and to sustain the vigilant monitoring and detection of IPV.

## Figures and Tables

**Table 1:** Information of sequencing data for IPV-positive *Macrobrachium rosenbergii* samples.

No.	Variant	GenBank Accession	Length	Virus Reads	FPKM	Library ID	Time	Location	Group
1	Infectious precocity virus 01/SEA/202305	PQ786402	12,630	633	9.57 × 10^2^	20230518002	202305	Southeast Asia	Previously Collected Samples
2	Infectious precocity virus 02/SEA/202401	PQ786403	12,630	113,059	3.00 × 10^5^	20240131030	202401	Southeast Asia	Previously Collected Samples
3	Infectious precocity virus 01/ZJ/202108	PP215336	12,519	10,280	1.14 × 10^4^	20210828001	202108	Zhejiang Province, China	Previously Collected Samples
4	Infectious precocity virus 01/ZJ/202111	PP215337	12,608	1,798,970	3.87 × 10^6^	20211103001	202108	Zhejiang Province, China	Previously Collected Samples
5	Infectious precocity virus 02/ZJ/202111	PP215338	12,519	1,112,410	2.30 × 10^6^	20211103002	202108	Zhejiang Province, China	Previously Collected Samples
6	Infectious precocity virus 01/SD/201901	PP215321	12,550	24,730	3.97 × 10^4^	20190123003-X	201901	Shandong Province, China	Previously Collected Samples
7	Infectious precocity virus 02/SD/201901	PP215322	12,559	24,078	4.74 × 10^1^	20190123006-O	201901	Shandong Province, China	Previously Collected Samples
8	Infectious precocity virus 03/SD/201901	PP215323	12,528	3788	6.40 × 10^3^	20190123006-X	201901	Shandong Province, China	Previously Collected Samples
9	Infectious precocity virus 04/SD/201901	PP215324	12,556	14,978	2.86 × 10^4^	20190123009-O	201901	Shandong Province, China	Previously Collected Samples
10	Infectious precocity virus 05/SD/201901	PP215325	12,535	285,626	6.26 × 10^5^	20190123011-O	201901	Shandong Province, China	Previously Collected Samples
11	Infectious precocity virus 01/ZJ/201806	PP054173	12,288	649,714	4.09 × 10^6^	A	201806	Jiangsu Province, China	Previously Collected Samples
12	Infectious precocity virus 01/JS/201906	PP215326	12,585	436,014	8.40 × 10^5^	20190602H1H2H3	201906	Jiangsu Province, China	Previously Collected Samples
13	Infectious precocity virus 02/JS/201906	PP215328	12,589	64,182	2.39 × 10^5^	20190602S3S4	201906	Jiangsu Province, China	Previously Collected Samples
14	Infectious precocity virus 01/JS/202008	PP215327	12,589	267,378	8.55 × 10^5^	20200821007	202008	Jiangsu Province, China	Previously Collected Samples
15	Infectious precocity virus 02/JS/202008	PP215329	12,554	71,914	1.60 × 10^5^	20200821008	202008	Jiangsu Province, China	Previously Collected Samples
16	Infectious precocity virus 03/JS/202008	PP215330	12,592	519,744	1.17 × 10^6^	20200821010	202008	Jiangsu Province, China	Previously Collected Samples
17	Infectious precocity virus 04/JS/202008	PP215331	12,588	162,836	3.75 × 10^5^	20200821011	202008	Jiangsu Province, China	Previously Collected Samples
18	Infectious precocity virus 05/JS/202008	PP215332	12,537	69,390	1.39 × 10^5^	20200821-tie10	202008	Jiangsu Province, China	Previously Collected Samples
19	Infectious precocity virus 06/JS/202008	PP054190	12,522	1872	3.93 × 10^3^	20200828005	202008	Jiangsu Province, China	Previously Collected Samples
20	Infectious precocity virus 07/JS/202008	PP054191	12,565	89,740	2.40 × 10^5^	20200828006	202008	Jiangsu Province, China	Previously Collected Samples
21	Infectious precocity virus 01/JS/202009	PP215333	12,544	3678	8.59 × 10^3^	20200929004	202009	Jiangsu Province, China	Previously Collected Samples
22	Infectious precocity virus 01/JS/202106	PP215334	12,582	272,778	6.12 × 10^5^	20210626-T1	202106	Jiangsu Province, China	Previously Collected Samples
23	Infectious precocity virus 02/JS/202106	PP215335	12,598	501,334	1.16 × 10^6^	20210626-T2	202106	Jiangsu Province, China	Previously Collected Samples
24	Infectious precocity virus 01/SH/202210	PQ786404	12,630	237	4.18 × 10^2^	J5_fastqc	202210	Shanghai, China	Public Database Samples
25	Infectious precocity virus 02/SH/202210	PQ786405	12,630	758	1.20 × 10^3^	J4_fastqc	202210	Shanghai, China	Public Database Samples
26	Infectious precocity virus ZJJS2019	ON382579.1	12,594	NA	NA	NA	201908	Zhejiang Province, China	Public Database Samples
27	Macrobrachium flavivirus 1 ZJHY201110	MT648663.1	12,628	NA	NA	NA	201110	Zhejiang Province, China	Public Database Samples
28	Macrobrachium flavivirus 1 JSYZ20170815	MT648664.1	12,628	NA	NA	NA	201708	Jiangsu Province, China	Public Database Samples
29	Infectious precocity virus MR2018	MT084113.1	12,630	NA	NA	NA	2018	China	Public Database Samples
30	Infectious precocity virus 01/JS/201806	PP210859	8789	608	8.03 × 10^−1^	T4	201806	Jiangsu Province, China	Previously Collected Samples
31	Infectious precocity virus 02/JS/201806	PP210860	3515	572	8.85 × 10^−1^	T3	201806	Jiangsu Province, China	Previously Collected Samples

## Data Availability

Newly generated sequences have been deposited in GenBank under accession numbers PQ786402–PQ786405. For the accession numbers of previously characterized IPV variants, refer to Table 1.

## Supplementary Materials

The following supporting information can be downloaded at: https://www.mdpi.com/article/10.3390/v17030425/s1, Table S1. Information of RNA-sequence database for *Macrobrachium rosenbergii* samples. Figure S1. Sequence identity of genome sequences and the amino acid polyproteins of the 29 IPV variants. The figure illustrates the pairwise sequence identity of the 29 IPV variants. The upper right section of the figure represents the sequence identity for genome sequences, while the lower left section details the sequence identity for amino acid polyproteins. Figure S2–S5. Multiple sequence alignment of domains and regions of the IPV variants from different geographical origins. Alignments were conducted using MAFFT across the following regions: trypsin-like peptidase domain in Figure S2, flavivirus DEAD domain and the DEXH-box helicase domain from NS3 protease helicase in Figure S3, C-terminal helicase domain of the viral helicase in Figure S4, and flavivirus RNA-directed RNA polymerase domain in Figure S5. The MR2018 sequence served as the consensus sequence. Identical amino acids are represented by “.” while differing amino acids are highlighted with different colored backgrounds. The names of the variants are positioned on the left side of each sequence, with amino acid positions indicated below each alignment block.
