# Supplementary material for: Surveillance and Genomic Evolution of Infectious Precocity Virus (IPV) from 2011 to 2024
Source: Viruses. 2025 Mar 15;17(3):425. doi: 10.3390/v17030425 (PMC11946579; doi:10.3390/v17030425)
Supplement: Supplementary file 1 [file viruses-17-00425-s001.zip › FigureS1Sequence identity of genome sequences and the amino acid polyproteins of 29 IPV variants.pdf]

Variant		No.1	No.2	No.3	No.4	No.5	No.6	No.7	No.8	No.9	No.10	No.11	No.12	No.13	No.14	No.15	No.16	No.17	No.18	No.19	No.20	No.21	No.22	No.23	No.24	No.25	No.26	No.27	No.28	No.29
01/SEA/202305	No.1		99.37	99.46	98.93	99.46	98.80	98.80	98.79	98.84	98.80	98.87	98.89	99.52	99.44	98.81	98.78	98.85	98.80	98.79	98.83	98.79	98.70	98.78	99.36	99.48	99.47	98.70	98.64	98.89
02/SEA/202401	No.2	99.58		99.48	98.79	99.48	98.66	98.65	98.65	98.69	98.66	98.71	98.74	99.55	99.46	98.67	98.64	98.71	98.66	98.65	98.69	98.65	98.56	98.64	99.26	99.49	99.49	98.55	98.50	98.74
01/ZJ/202108	No.3	99.71	99.76		98.84	100	98.72	98.72	98.71	98.76	98.72	98.77	98.80	99.94	99.77	98.74	98.70	98.75	98.72	98.73	98.75	98.71	98.62	98.72	99.53	99.80	99.80	98.80	98.77	98.79
01/ZJ/202111	No.4	99.49	99.49	99.62		98.84	99.86	99.86	99.86	99.91	99.86	99.95	99.94	98.91	98.83	99.86	99.83	99.90	99.85	99.84	99.87	99.84	99.76	99.85	99.29	98.91	98.86	99.86	99.76	99.95
02/ZJ/202111	No.5	99.71	99.76	100	99.62		98.72	98.72	98.71	98.76	98.72	98.77	98.80	99.94	99.77	98.74	98.70	98.75	98.72	98.73	98.75	98.71	98.62	98.72	99.53	99.80	99.80	98.80	98.77	98.79
01/SD/201901	No.6	99.36	99.35	99.49	99.87	99.49		100	99.99	99.78	99.94	99.82	99.83	98.79	98.71	99.74	99.71	99.79	99.73	99.74	99.75	99.72	99.69	99.74	99.16	98.78	98.73	99.80	99.71	99.82
02/SD/201901	No.7	99.36	99.35	99.49	99.87	99.49	100		99.99	99.78	99.94	99.82	99.82	98.78	98.70	99.74	99.70	99.79	99.73	99.72	99.75	99.72	99.68	99.74	99.16	98.77	98.73	99.81	99.72	99.81
03/SD/201901	No.8	99.36	99.35	99.49	99.87	99.49	100	100		99.77	99.93	99.82	99.82	98.78	98.70	99.73	99.70	99.78	99.72	99.73	99.74	99.71	99.68	99.74	99.15	98.77	98.72	99.78	99.70	99.81
04/SD/201901	No.9	99.39	99.38	99.51	99.89	99.51	99.76	99.76	99.76		99.78	99.95	99.85	98.82	98.74	99.78	99.74	99.81	99.77	99.78	99.78	99.75	99.67	99.76	99.21	98.81	98.77	99.88	99.74	99.96
05/SD/201901	No.10	99.41	99.41	99.54	99.92	99.54	99.95	99.95	99.95	99.81		99.82	99.83	98.79	98.71	99.74	99.70	99.79	99.73	99.74	99.75	99.72	99.69	99.73	99.16	98.78	98.73	99.82	99.75	99.82
01/ZJ/201806	No.11	99.47	99.46	99.60	99.97	99.60	99.84	99.84	99.84	99.92	99.89		99.89	98.84	98.75	99.81	99.78	99.85	99.81	99.81	99.83	99.80	99.71	99.80	99.24	98.84	98.78	99.86	99.72	99.99
01/JS/201906	No.12	99.47	99.46	99.60	99.95	99.60	99.81	99.81	99.81	99.84	99.87	99.92		98.87	98.79	99.81	99.78	99.86	99.80	99.79	99.82	99.79	99.73	99.80	99.25	98.86	98.82	99.81	99.71	99.89
02/JS/201906	No.13	99.74	99.78	99.97	99.65	99.97	99.51	99.51	99.51	99.54	99.57	99.62	99.62		99.83	98.80	98.77	98.82	98.79	98.78	98.81	98.78	98.69	98.79	99.59	99.86	99.86	98.84	98.79	98.86
01/JS/202008	No.14	99.71	99.76	99.89	99.62	99.89	99.49	99.49	99.49	99.51	99.54	99.60	99.60	99.92		98.72	98.69	98.74	98.71	98.70	98.73	98.70	98.61	98.71	99.48	99.86	99.97	98.76	98.71	98.78
02/JS/202008	No.15	99.47	99.46	99.60	99.97	99.60	99.84	99.84	99.84	99.87	99.89	99.95	99.92	99.62	99.60		99.89	99.77	99.92	99.92	99.94	99.90	99.63	99.91	99.17	98.79	98.74	99.82	99.74	99.81
03/JS/202008	No.16	99.44	99.43	99.57	99.95	99.57	99.81	99.81	99.81	99.84	99.87	99.92	99.89	99.60	99.57	99.92		99.74	99.96	99.94	99.95	99.92	99.60	99.93	99.14	98.76	98.71	99.71	99.63	99.78
04/JS/202008	No.17	99.39	99.38	99.51	99.89	99.51	99.76	99.76	99.76	99.78	99.81	99.87	99.84	99.54	99.51	99.87	99.84		99.76	99.75	99.79	99.75	99.69	99.76	99.20	98.82	98.77	99.83	99.74	99.85
05/JS/202008	No.18	99.49	99.49	99.62	100	99.62	99.87	99.87	99.87	99.89	99.92	99.97	99.95	99.65	99.62	99.97	99.95	99.89		99.98	99.98	99.94	99.63	99.95	99.16	98.78	98.73	99.81	99.73	99.80
06/JS/202008	No.19	99.49	99.49	99.62	100	99.62	99.87	99.87	99.87	99.89	99.92	99.97	99.95	99.65	99.62	99.97	99.95	99.89	100		99.97	99.95	99.62	99.96	99.15	98.77	98.72	99.80	99.72	99.79
07/JS/202008	No.20	99.49	99.49	99.62	100	99.62	99.87	99.87	99.87	99.89	99.92	99.97	99.95	99.65	99.62	99.97	99.95	99.89	100	100		99.97	99.65	99.98	99.19	98.81	98.76	99.80	99.72	99.82
01/JS/202009	No.21	99.47	99.46	99.60	99.97	99.60	99.84	99.84	99.84	99.87	99.89	99.95	99.92	99.62	99.60	99.95	99.92	99.87	99.97	99.97	99.97		99.62	99.94	99.15	98.77	98.72	99.78	99.70	99.79
01/JS/202106	No.22	99.39	99.38	99.51	99.89	99.51	99.76	99.76	99.76	99.78	99.81	99.87	99.84	99.54	99.51	99.87	99.84	99.78	99.89	99.89	99.89	99.87		99.63	99.06	98.68	98.63	99.68	99.61	99.71
02/JS/202106	No.23	99.49	99.49	99.62	100	99.62	99.87	99.87	99.87	99.89	99.92	99.97	99.95	99.65	99.62	99.97	99.95	99.89	100	100	100	99.97	99.89		99.14	98.76	98.74	99.79	99.71	99.78
01/SH/202210	No.24	99.68	99.70	99.89	99.73	99.89	99.60	99.60	99.60	99.62	99.65	99.70	99.70	99.92	99.84	99.70	99.68	99.62	99.73	99.73	99.73	99.70	99.62	99.73		99.56	99.51	99.07	98.97	99.26
02/SH/202210	No.25	99.71	99.76	99.89	99.68	99.89	99.54	99.54	99.54	99.57	99.60	99.65	99.65	99.92	99.95	99.65	99.62	99.57	99.68	99.68	99.68	99.65	99.57	99.68	99.89		99.89	98.67	98.62	98.86
▲ZJJS2019	No.26	99.74	99.78	99.92	99.65	99.92	99.51	99.51	99.51	99.54	99.57	99.62	99.62	99.95	99.97	99.62	99.60	99.54	99.65	99.65	99.65	99.62	99.54	99.65	99.87	99.97		98.73	98.68	98.81
▲ZJHY201110	No.27	99.44	99.43	99.57	99.95	99.57	99.81	99.81	99.81	99.84	99.87	99.92	99.89	99.60	99.57	99.92	99.89	99.84	99.95	99.95	99.95	99.92	99.84	99.95	99.68	99.62	99.60		99.81	99.75
▲JSYZ20170815	No.28	99.47	99.46	99.60	99.97	99.60	99.84	99.84	99.84	99.87	99.89	99.95	99.92	99.62	99.60	99.95	99.92	99.87	99.97	99.97	99.97	99.95	99.87	99.97	99.70	99.65	99.62	99.92		99.60
▲MR2018	No.29	99.47	99.46	99.60	99.97	99.60	99.84	99.84	99.84	99.92	99.89	100	99.92	99.62	99.60	99.95	99.92	99.87	99.97	99.97	99.97	99.95	99.87	99.97	99.70	99.65	99.62	99.92	99.95	
