# Supplementary material for: Surveillance and Genomic Evolution of Infectious Precocity Virus (IPV) from 2011 to 2024
Source: Viruses. 2025 Mar 15;17(3):425. doi: 10.3390/v17030425 (PMC11946579; doi:10.3390/v17030425)
Supplement: Supplementary file 1 [file viruses-17-00425-s001.zip › FigureS4The C-terminal helicase domain of viral helicase.pdf]

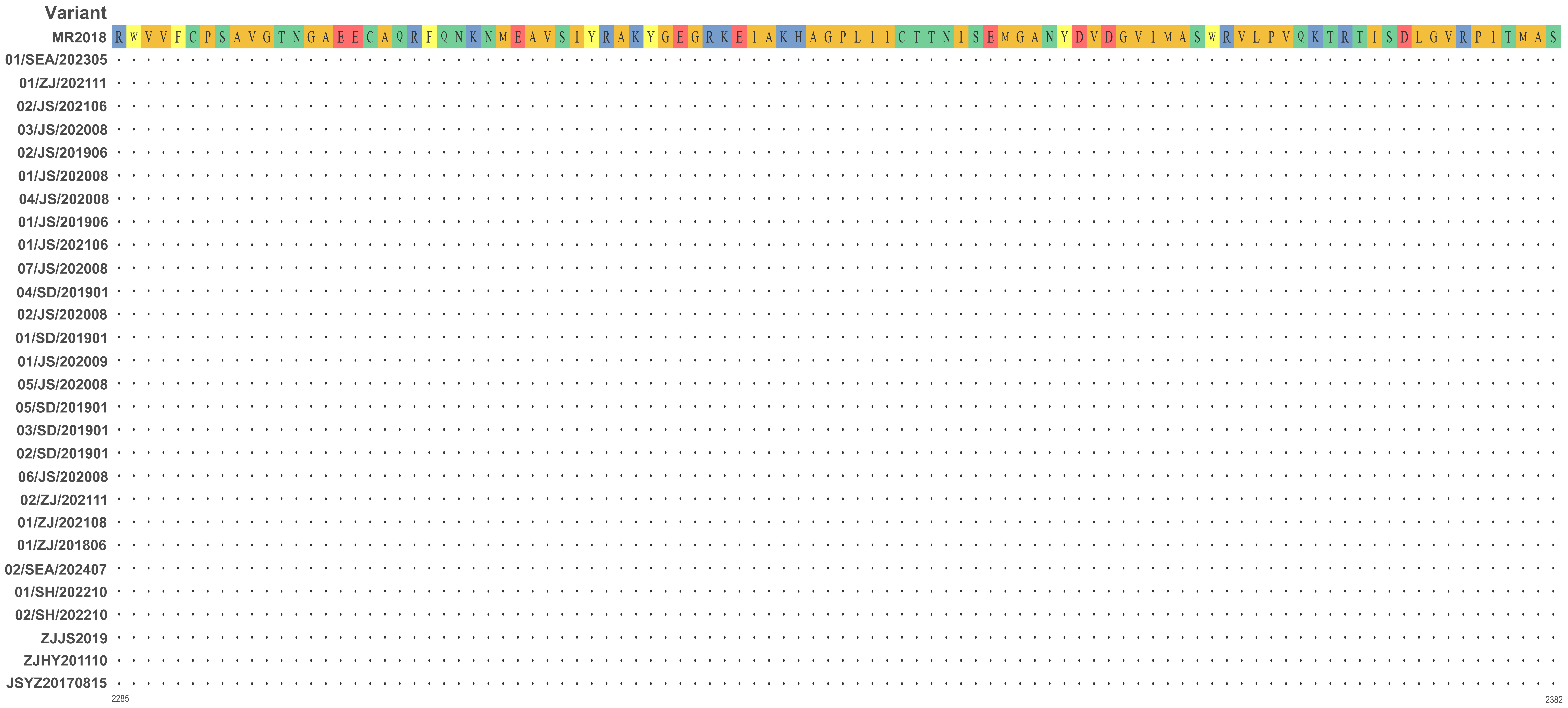
Variant
MR2018
RWVVFCPSAVGTNGAEECAQRFQNKNMEAVSIYRAKYGEGRKEIAKHAGPLIICTTNISEMGANYDVDGVIMASWRVLPVQKTRTISDLGVRPITMAS
01/SEA/202305
01/ZJ/202111
02/JS/202106
03/JS/202008
02/JS/201906
01/JS/202008
04/JS/202008
01/JS/201906
01/JS/202106
07/JS/202008
04/SD/201901
02/JS/202008
01/SD/201901
01/JS/202009
05/JS/202008
05/SD/201901
03/SD/201901
02/SD/201901
06/JS/202008
02/ZJ/202111
01/ZJ/202108
01/ZJ/201806
02/SEA/202407
01/SH/202210
02/SH/202210
ZJJS2019
ZJHY201110
JSYZ20170815
2285
2382
